# Recent progress in studies of photocages

**DOI:** 10.1002/smo.20220003

**Published:** 2023-03-20

**Authors:** Yajing Li, Maolin Wang, Fang Wang, Sheng Lu, Xiaoqiang Chen

**Affiliations:** ^1^ State Key Laboratory of Materials‐Oriented Chemical Engineering College of Chemical Engineering Jiangsu National Synergetic Innovation Center for Advanced Materials (SICAM) Nanjing Tech University Nanjing China

**Keywords:** photo‐controlled release, photocage, photoprotecting group, photoremoveable protecting group

## Abstract

Photocages are a class of substances containing photosensitive groups, also known as “photoremovable protecting groups”, from which target substances are released upon exposure to specific wavelengths of light. The substances released in the light‐promoted processes have chemical or biological properties that enable them to carry specifically designed functions. As a result, photocages can be utilized in various fields such as chemistry, biology, and medicine. In this paper, progress made in research carried out in recent years aimed at developing photocage molecules with different photosensitive moieties is reviewed.

## Graphical abstract

Photocages are a group of promising substances which mainly consist of photosensitive groups and release specific molecules under light irradiation. This review summarizes the recent photocages with different photosensitive groups and proposes an outlook for the design of photocages.

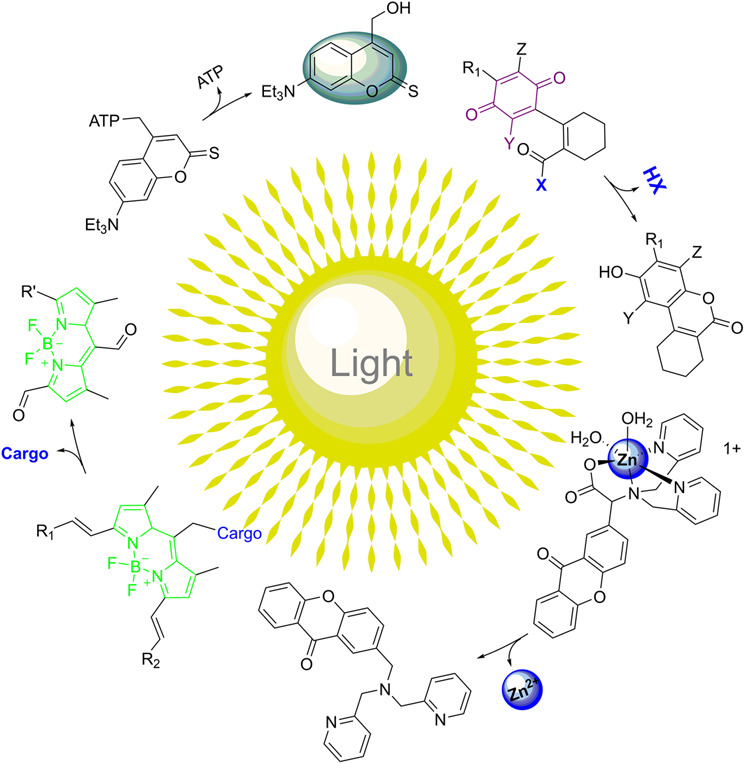

## 1 INTRODUCTION

Using light to promote controlled release of substances has several advantageous features, including (i) that light is clean and inexpensive energy that has accessibility to many locations,^[1]^ (ii) light promoted reactions are more selective than those that are thermally activated, (iii) the controllability of light reaction is high so that the rate of target release can be governed by simply varying the intensity and wavelength, (iv) light reactions have a wide range of applications as a consequence of the fact that they are most often temperature independent, and (v) long wavelength light is both less damaging to cells and more penetrable into cells, tissues, and other organisms.^[2]^

Photocages are photosensitive substances that release target products upon light irradiation. In 1978, Hoffmann et al. were the first to propose the “cage” concept and to synthesize the earliest photocage, “Caged ATP”,^[3]^ containing 2‐nitrobenzyl moiety as the photosensitive group and adenosine triphosphate (ATP) as the target product that is released by using 340 nm light irradiation. They demonstrated that “Caged ATP” is not degraded by intracellular ATPases and that photo‐released ATP effectively activates the Na:K pump in a cell. Photocage molecules consist of two basic components including a photosensitive group photoremovable protecting group (PPG) and a specific target substance that is released upon light irradiation. In the cages, targets are physically and chemically protected by the PPG, which undergoes photoreaction that releases the target.^[4]^ As a result, targets do not function until they are released at specific locations where their properties are restored. Various high‐performance photocages have been developed during the nearly half a century since they were first conceptualized.^[5]^ Many photosensitive groups have been developed since that time, including *o*‐nitrobenzyl derivatives,^[6]^ coumarin derivatives,^[7]^ BODIPY,^[8]^ xanthene derivatives,^[9]^ quinone and diarylenes derivatives,^[10]^ and others.^[11]^

Photocages are utilized mainly in chemicals, materials, and biological fields for applications that include photolithographic fabrication of gene chips,^[12]^ photo‐responsive organic materials and polymers,^[13]^ and protecting groups for use in multistep organic synthesis. Especially in the biological field, photocages have been used to generate a variety of substances in biological microenvironments including proteins, nucleotides, ions, neurotransmitters, drugs, fluorescent dyes, and small biological molecules. This review describes some recently developed photocages in the six categories listed above and the progress that has been made in controlling the release of active substances.

## 2 O‐NITROBENZYL‐BASED PHOTOCAGES

The non‐fluorescent *o*‐nitrobenzyl moiety is a common photoprotective group that is occasionally used as a fluorescence quencher. Owing to their confirmed photolytic mechanism and excellent photolytic efficiency, increasing numbers of *o*‐nitrobenzyl‐based photocages have been prepared and used in a wide range of chemical and biological fields.

**FIGURE 1:**
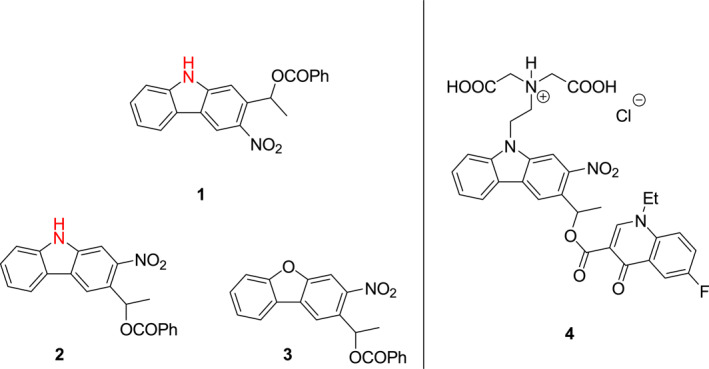
Photocages **1**–**4**.

Martin et al. designed two novel carbazole‐based *o*‐nitrobenzyl skeletons **1** and **2** (Figure 1).^[15]^ In contrast to the previously studied dibenzofuran ring containing photocage **3**, the nitrogen center in the carbazole rings in photocages **1** and **2** causes them to have redshifted absorption wavelength maxima, higher water solubilities, and it serves as a site for the introduction of functional groups. The efficiencies of photolytic reactions of **1** and **2** upon irradiation at 390 nm are 40‐ and 25‐fold higher, respectively, than that of photocage **3** irradiated at the same wavelength. Moreover, the respective photolysis efficiencies of photocages **1** and **2** are 150‐ and 20‐fold higher than that of photocage **3** when irradiated using 400 nm light. In addition, guided by these observations they designed and constructed photocage **4**, which releases a fluoroquinolone antibiotic under 400 nm light that significantly inhibits the growth of *Escherichia coli.*

**FIGURE 2:**
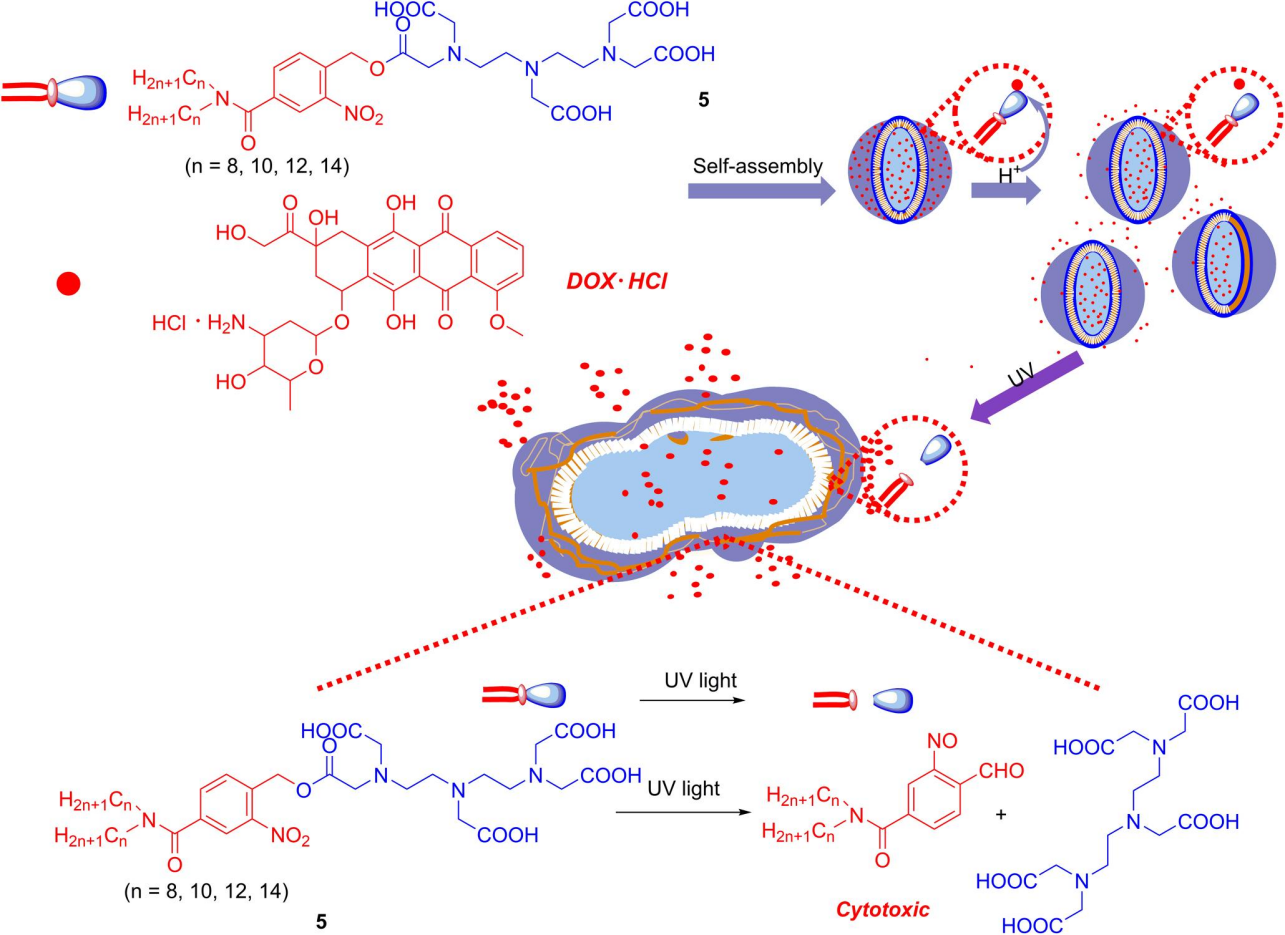
Mechanism of drug release in an acidic environment and with UV irradiation of DOX‐loaded liposomes in MCF‐7 cells and the photolysis mechanism of photocage **5**.

Qiao et al. developed amphiphilic photocage **5** with good biocompatibility and low cytotoxicity.^[16]^ This substance contains the hydrophilic diethylenetriaminepentaacetic acid (DTPA) group and hydrophobic double long carbon chains linked through an *o*‐nitrobenzyl moiety, and it can release nitrosobenzaldehyde and DTPA after UV light irradiation (Figure 2). In addition, the liposomes made from photocage **5** have good stability and can also be photolyzed by UV light to produce nitrosobenzene products to inhibit the proliferation of cancer cells. Notably, these liposomes have high drug encapsulation capacity owing to the strong electrostatic interactions between the multiple carboxyl groups and the amino groups of drug molecules, along with the enlarged hydrophilic cavity formed by the ones with longer hydrophobic chains. The authors further demonstrated the encapsulation of doxorubicin (DOX) into the liposomes prepared by photocage **5** to form the pH/photo dual‐responsive DOX‐loaded liposomes. These liposomes can enter into endosomes/lysosomes (pH 4.0–6.0) of the MCF‐7 cells and release part of the DOX molecules in response to the acidic environment while releasing the majority of the DOX and other photolysis products under UV irradiation, resulting in higher anticancer activity than DOX alone.

**FIGURE 3:**
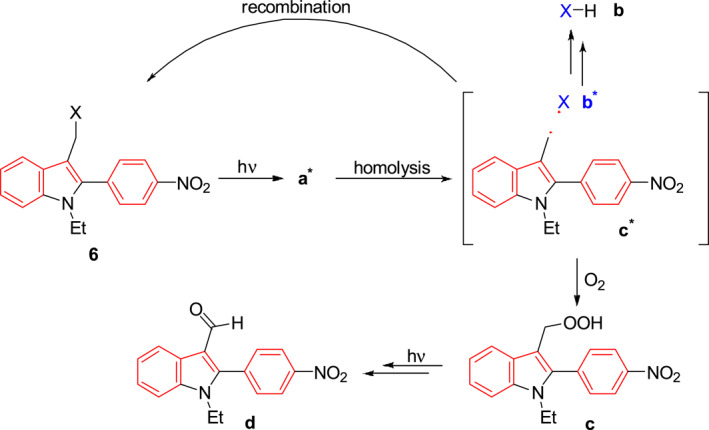
Proposed mechanism of the photochemical decomposition of photocage **6**.

Abe et al. developed the novel, low cytotoxic, thermally stable photocage **6** which contains a 2‐(4‐nitrophenyl)‐1H‐indolyl‐3‐methyl chromophore (Figure 3).^[17]^ Upon 405 nm irradiation, photocage **6** is transformed to its singlet excited state a* which undergoes homolysis to produce the radical pair of b* and c*. Under the environment with the proton source, the radical b* combines with H to produce b while the other radical c* can be oxidized to obtain compound c, which undergoes further photolysis to produce the compound d. Owing to its low cytotoxicity, photocage **6** could be useful in further biological studies.

**FIGURE 4:**
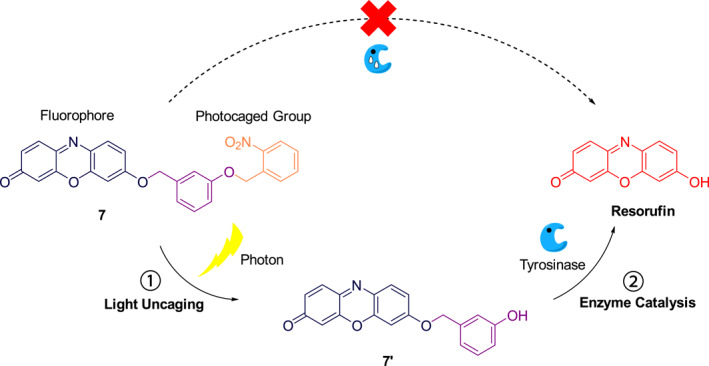
Mechanism of detection of tyrosinase by photoactivation of photocage **7**.

Yang et al. devised the sequential light and tyrosinase (TYR) activated photocage **7** by linking the TYR probe **7′** to the photolabile *o*‐nitrobenzyl group (Figure 4).^[18]^ Photocage **7** is inactive to TYR because the *o*‐nitrobenzyl causes steric hindrance. Upon irradiation with 365 nm UV light, the *o*‐nitrobenzyl is cleaved to uncage the probe **7′** that has restored activity toward TYR associated with a color change from colorless to pink. When probe **7′** contacts with TYR, it will release the bright red fluorescing resorufin. Experimental studies have shown that photocage **7** has the ability to generate the endogenously active tyrosinase probe upon irradiation in an intracellular environment, demonstrating that it is a promising molecular imaging tool for studying tyrosinase‐related physiological functions and pathological effects. Furthermore, this sequential activation strategy has great potential for the development of photo‐controllable enzymatic fluorogenic probes with spatiotemporal resolution.

**FIGURE 5:**
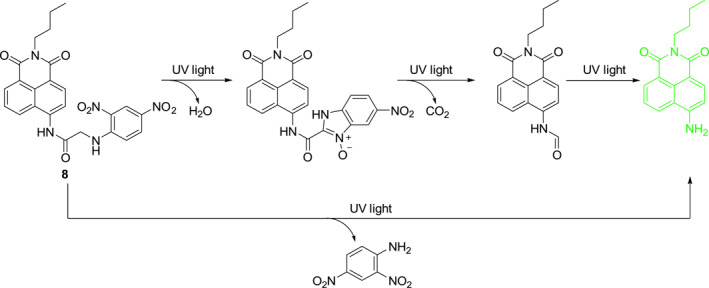
Structure of photocage **8** and the possible photolysis mechanism upon exposure to UV light.

Chen et al. developed the novel photocage **8** containing glycine‐linked naphthalimide and *o*‐nitrobenzyl groups (Figure 5).^[19]^ Upon irradiation, **8** releases a fluorescent naphthalimide derivative through different routes shown in Figure 5. The released fluorescence intensity is linearly correlated with the intensity of UV irradiation. In addition, the authors prepared a portable electrospun fiber by embedding **8** in polyethylene oxide (PEO). Experiments show that this nanofiber has good capability for UV quantitative detection and can also reflect the solar UV intensity well.

## 3 COUMARIN‐BASED PHOTOCAGES

Coumarins were identified as secondary metabolites in a variety of plants in 1820. In 1984, Givens and Matuszewski discovered that phosphate is released from (coumarin‐4‐yl)‐methyl upon light irradiation.^[20]^ Since that time, coumarin has been broadly used as a photoprotective moiety. Coumarin‐based photocages have a wide variety of advantages, such as high molar absorption coefficients, high photolysis efficiencies, and rapid photolysis rates. Moreover, the spectroscopic and photochemical properties of these substances can be readily tuned by changing substituents on the coumarin ring. As a result, coumarin and related derivatives have been increasingly used in the chemical and biomedical fields.^[21]^

**FIGURE 6:**
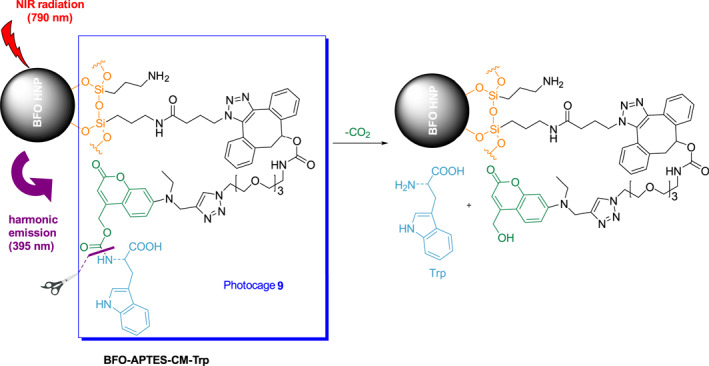
The structure of BFO‐APTES‐CM‐Trp and schematic of second harmonic emission upon 790 nm irradiation and tryptophan release.

Amino acids are an essential group of substances in living systems and are involved in tissue metabolism, growth, maintenance, and repair.^[22]^ Amino acids play important roles in maintaining the physiological functions and life processes of humans. Owing to their biological importance, amino acids have received significant attention in elucidating the roles they play in living organisms and biological processes. In 2019, Gerber‐Lemaire et al. successfully constructed the near‐infrared light‐triggered system, BFO‐APTES‐CM‐Trp, in which bismuth ferrite harmonic nanoparticles (BFO HNPs) are linked to photocage **9** (Figure 6).^[23]^ Upon irradiation at 790 nm, BFO‐APTES‐CM‐Trp emits light at the second harmonic wavelength 395 nm and releases tryptophan from photocage **9**. Experiments have demonstrated that photocage **9** and its cleavage products are not toxic to cells. Since the photolysis mechanisms of coumarin‐4‐yl methoxy carbonyl derivatives should function to release amines, alcohols, thiols, and carboxylic acids,^[24]^ it should be possible to couple various molecular entities, including drugs, to BFO HNPs to create photocages for both deep tissue imaging and release of therapeutics.

**FIGURE 7:**
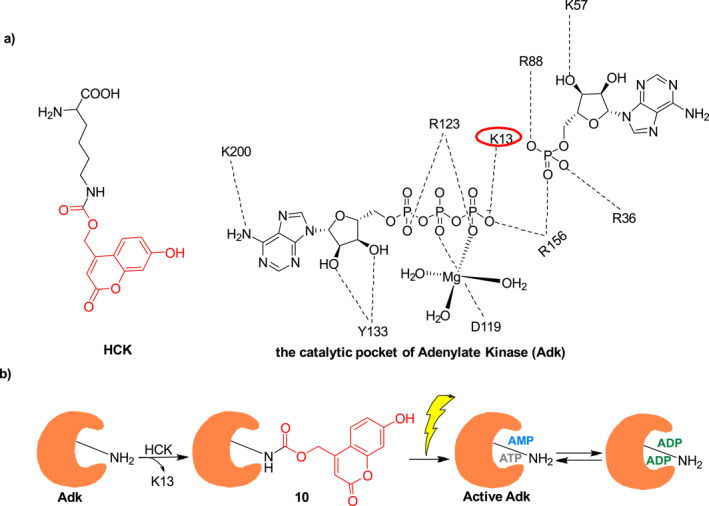
(a) The structure of HCK and the active site K13 of Adk; (b) Diagram of light regulation ATP using photocage **10**.

Enzymes are proteins or nucleic acids that catalyze processes in living organisms that are essential for metabolism. In 2020, Deiters et al. described photocage **10** (Figure 7) for photo‐initiated release of the enzyme adenylate kinase (Adk).^[25]^ In this system, photosensitive hydroxycoumarin is covalently linked to Adk by using the modified lysine derivative HCK superimposed to the position of K13 in the active site of Adk (Figure 7a) to form **10**, which does not have Adk activity. Upon UV irradiation of photocage **10**, the photosensitive hydroxycoumarin group cleaved to Adk enzyme activity was restored by lysine residue rapidly interacting with ATP and adenosine monophosphate (AMP) to produce adenosine diphosphate (ADP). Photocage **10** can be employed to rapidly consume intracellular ATP, thereby accelerating mammalian cell growth. What's more, caging of Adk in photocage **10** provides a new route for light‐regulated direct perturbation of cellular ATP concentrations, which should enable studies of ATP‐coupled physiological events in a temporal manner.

**FIGURE 8:**
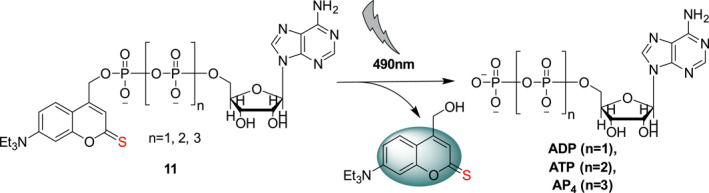
Photo‐uncaging schematic of photocage **11**.

In 2020, Jessen et al. designed and synthesized photocage **11** (Figure 8), which contains 7‐diethylamino‐4‐hydroxymethyl‐thiocoumarin (thio‐DEACM) as the photosensitive group.^[26]^ Under the irradiation of 490 nm light, photocage **11** can be used to release the adenosine phosphates ADP, ATP, and AP_4_. Moreover, the thio‐DEACM photocage **11** has better red‐shifted absorption maximum and greater photolysis efficiency compared with those of the conventional 7‐diethylamino‐4‐hydroxymethyl‐coumarin cage group. Replacement of O with S improved the photolytic properties of the coumarin scaffold and also stability in aqueous solutions. Thus, it is possible to install thio‐DEACM on phosphate‐containing signaling molecules in live cell studies.

Photosensitive groups have become more widely used in the field of photo‐pharmacy due to increasing demand for remote, spatiotemporally controlled drug release. Two main factors influence the efficiency of payload release from PPGs, including (i) the ability of the PPG to absorb photons at the irradiation wavelength (represented by the molar attenuation coefficient *ε*, M^−1^ cm^−1^) and (ii) the quantum yield (φ, QY) of the photochemical uncaging process. Significant progress has been made in recent years with respect to the first issue, but the enhancement of quantum efficiencies remains challenging.

**FIGURE 9:**
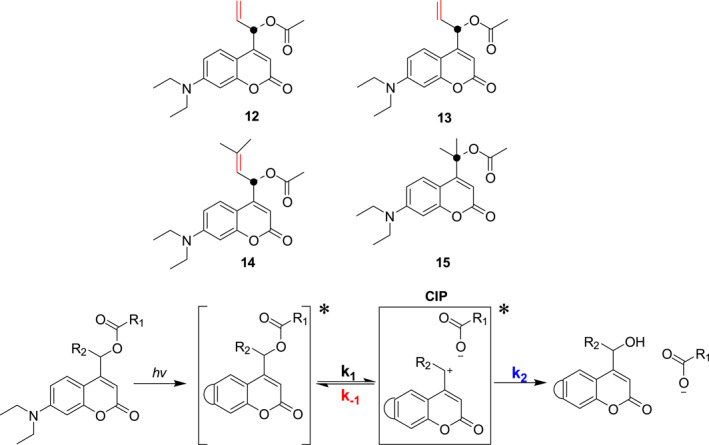
The structures of photocages **12**–**15** and schematic of the coumarin photocleavage mechanism.

In 2022, Feringa et al. devised a strategy to increase quantum yields by stabilizing the intermediate cation generated in the photolytic process.^[27]^ In this work, the four photocages **12–15** having coumarin as the photosensitive group (Figure 9) were designed and synthesized. Allyl substitution in photocages **12–14** promotes cation stabilization that leads to an increase in the energy potential for CIP (Contact Ion Pair) recombination more than super‐conjugation in tertiary coumarin **15**, and the quantum efficiency for photocleavage of allyl‐substituted coumarin photocage **14** is 27%, a value that is 16‐fold higher than that of the unsubstituted coumarin model. Furthermore, the strategy for increasing PPG efficiency by cation stabilization is versatile for the release of a variety of targets, including carboxylic acids and amines, as well as the use of different PPGs such as coumarin and BODIPY, which will aid in the development of efficient photocages for a wide range of applications.

## 4 BODIPY‐BASED PHOTOCAGES

Owing to its narrow absorption band and good biocompatibility, the boron difluoride complex of a dipyrromethene‐termed BODIPY has become a promising candidate for visible light photo‐caging. BODIPY photocages, first developed by Winter and Weinstain, have good photochemical reaction efficiencies and membrane permeabilities. In recent years, studies conducted on BODIPY‐based photocages containing sulfonate modification, π‐extension, and structural optimization have led to the development of many novel photocages having adjustable water solubilities, tunable membrane permeabilities, and large quantum yields that have been utilized in a wider range of applications.^[28]^

**FIGURE 10:**
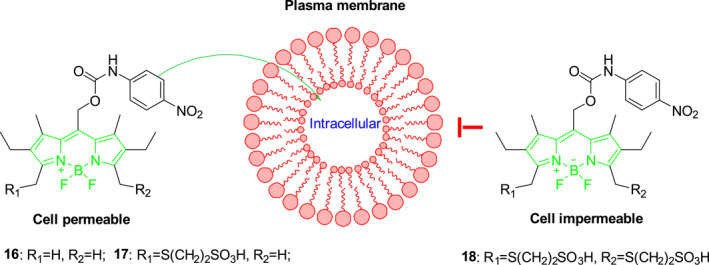
The structures of photocages **16**, **17**, and **18** and their mechanism of action.

Weinstain et al. designed water‐soluble BODIPY photocages **16–18** using a peripheral sulfonate‐controlled cellular localization strategy (Figure 10).^[29]^ These photocages, having improved water solubilities, are created by linking alkyl sulphonate groups to the BODIPY core through a thioether bond while retaining the photoreaction properties and favorable spectroscopic characteristics of the parent. Photocages **16–18** undergo photolysis upon being irradiated with 545 nm light. In contrast to photocages without the sulphonic acid group (**16)** and with only one sulphonic acid group **(17)** readily passing through the cell membrane, the analog photocage **18** with two sulphonic acid groups has difficulty passing through cell membranes. Thus, the disulfonated BODIPY photocage is potential for use in the modulation of extracellular proteins and cell‐surface receptors while monosulfonated BODIPY photocages can modulate intracellular targets.

**FIGURE 11:**
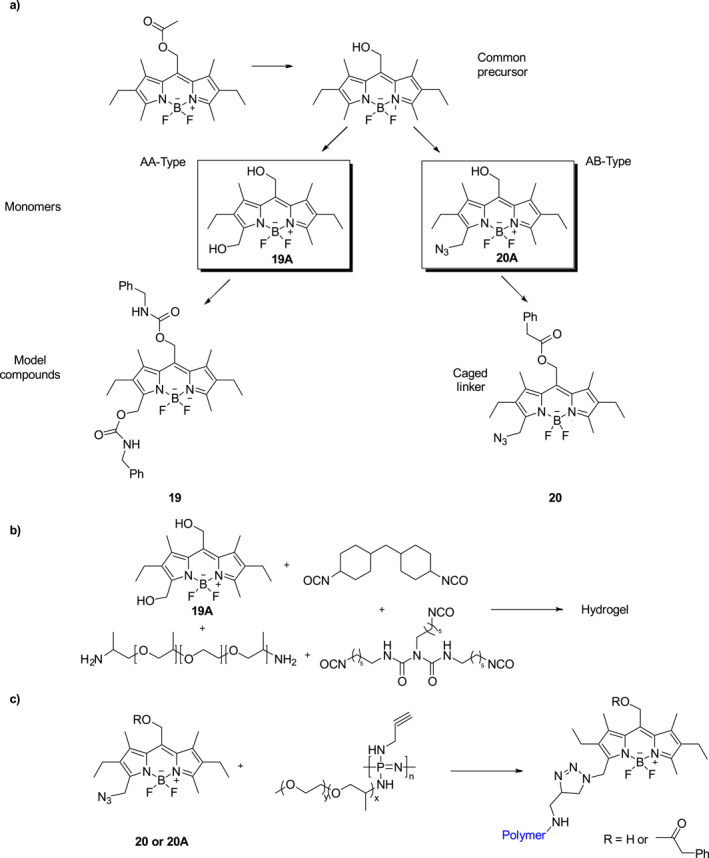
(a) Pathways for preparation of bifunctional and hetero‐bifunctional BODIPY monomers **19A** and **20A** and the synthesis of the respective small model compounds **19** and **20**, (b) Constituents of the synthesized BODIPY‐hydrogel, and (c) Scheme for reaction of BODIPY **20** or **20A** with poly (organo) phosphazene.

Teasdale et al. described the respective AA‐type and AB‐type bifunctional BODIPY monomers **19A** and **20A**, which were incorporated into the corresponding small molecule model compounds **19** and **20** (Figure 11a).^[30]^ They can be photolyzed upon the irradiation of green light. In addition, the authors employed monomer **19A** as a photocleavable linker in a pale purple color hydrogel (Figure 11b) that undergoes 365 nm light promoted photolysis to release green fluorescence. Furthermore, the hydrogel completely dissolves by photocleavage of the BODIPY linkages induced using long‐term irradiation at 365 nm. Monomer **20A** could act as a photocleavable linker in potential water‐soluble degradable polymeric photocages. The small molecule model photocage **20** was prepared and subsequently decorated with phenylacetic acid and incorporated into a polymer (Figure 11c), which undergoes controlled release of the phenylacetic acid upon 505 nm irradiation. Thus, BODIPY‐based photosensitive materials can be used in degradable polymers, sacrificial materials for lithography, and for the delivery of drugs.

**FIGURE 12:**
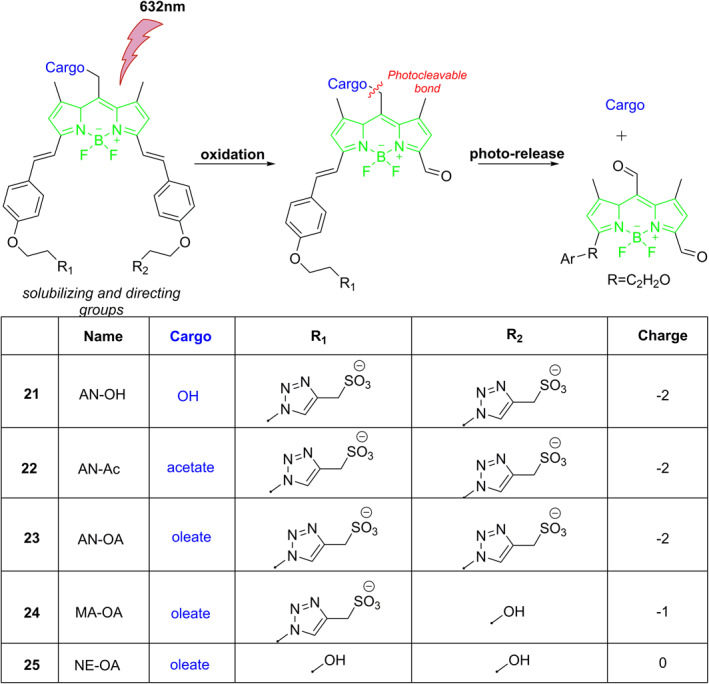
The structures of developed BODIPY cages and their mechanism of action.

Slanina et al. described photocages **21–25** that have a π‐extended BODIPY core (Figure 12).^[31]^ The electron‐donating alkoxy substituents in these substances cause the absorption maximum to undergo a red shift from 660 to 700 nm. The release of target cargo can be described as a two‐step photoreaction. Initially, the photocage molecules are oxidized to corresponding aldehydes and followed by secondary photolysis to release the target cargo and oxidation products under the irradiation of 632 nm red light. More intriguingly, because these photocages have water solubilities and membrane permeability that can be varied by changing the number of charged sulfonate groups, it is possible to adjust their specific locations in HeLa cells.

**FIGURE 13:**
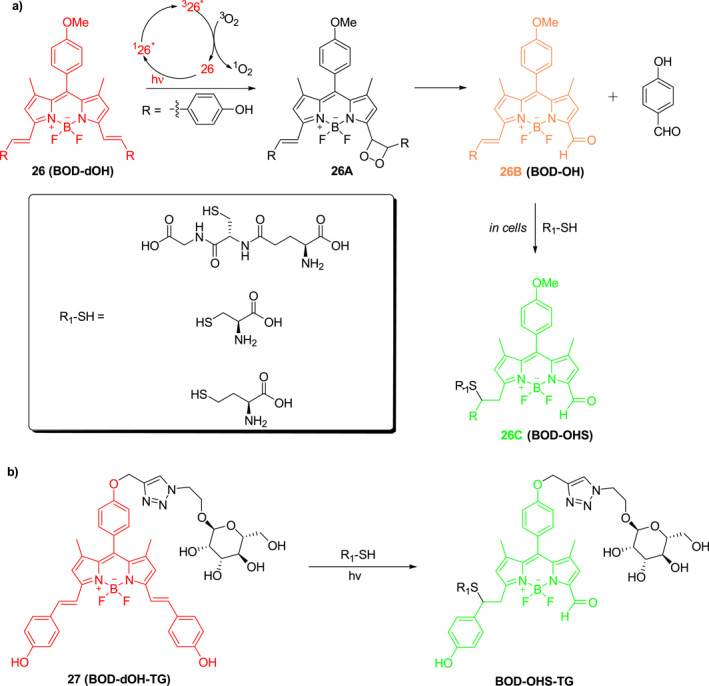
The structures and mechanism of action of photocages **26** and **27**.

Geng et al. developed a red fluorescent BODIPY skeleton **26** that can be oxidatively cleaved to release aldehyde (Figure 13a).^[32]^ Photo‐activated 26* by 630 nm light leads to the generation of ^1^O_2_, which can react with **26** via cycloaddition to form the thermally labile intermediate product **26A** and finally rearrange to give **26B**. The **26B** was shown to readily react with thiol groups in HeLa cells to form adduct **26C**, accompanied by a fluorescence emission shift from 673 to 548 nm (*λ*_ex_ = 488 nm), demonstrating the potential for thiol detection in living cells. In addition, photocage **27** (BOD‐dOH‐TG), a mannose functionalized derivative of **26** was developed by the authors to serve as a red fluorescing photocage (Figure 13b) for the detection of thiols in human hepatoma HepG2 by the production of the green fluorescing BOD‐OHS‐TG under 630 nm light excitation.

**FIGURE 14:**
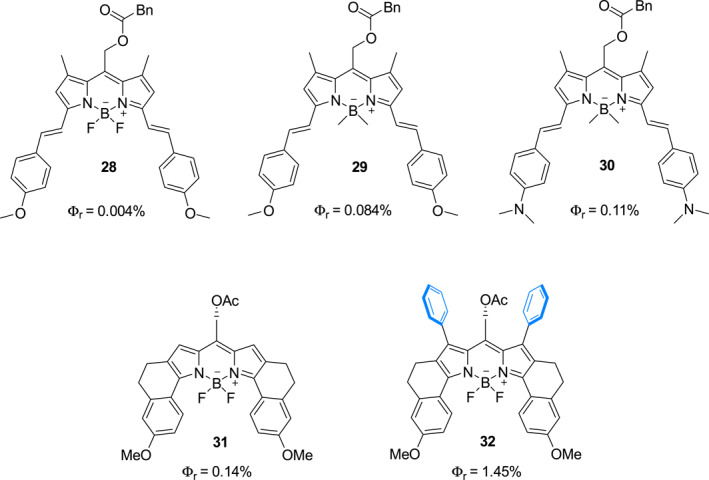
The structures of photocages **28–32**.

Winter et al. described a strategy to effectively increase the quantum yield of BODIPY‐based photocages by blocking unproductive conical intersections (Figure 14).^[33]^ They found that the ring‐fused BODIPY photocage **31** has a 0.14% quantum efficiency that is almost 35‐fold higher than that of **28** (Φ_*r*_ = 0.04%) and a higher quantum yield than those of photocages **29** (Φ_*r*_ = 0.08%) and **30** (Φ_*r*_ = 0.11%), which were probed in earlier studies.^[34]^ Moreover, the conformationally restrained BODIPY photocage **32** has a quantum yield (Φ_*r*_ = 1.45%) that is almost 10‐fold higher than that of photocage **31**.

## 5 XANTHENE‐BASED PHOTOCAGES

Xanthene is the basic skeleton of a large class of fluorescent substances, dyes, and synthetic drugs. Photocages based on xanthene and their derivatives have been applied to the detection of metal ions and photodynamic therapy in recent years.^[35]^

**FIGURE 15:**
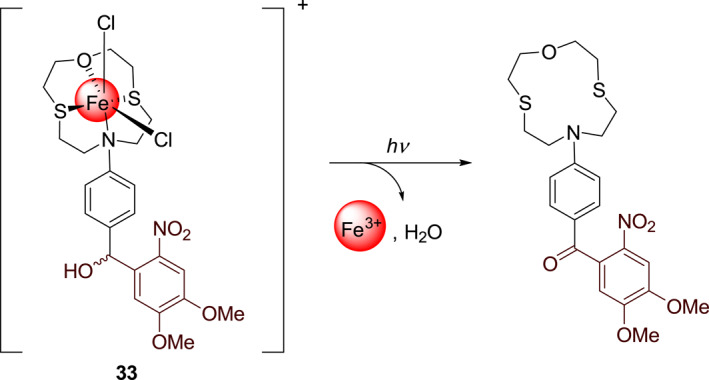
The structure of photocage **33** and its mechanism of photo‐controlled release Fe^3+^.

**FIGURE 16:**
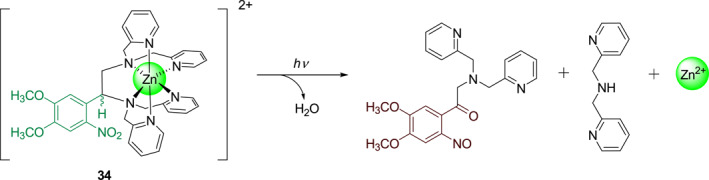
The structure of photocage **34** and its mechanism of photo‐controlled release Zn^2+^.

**FIGURE 17:**
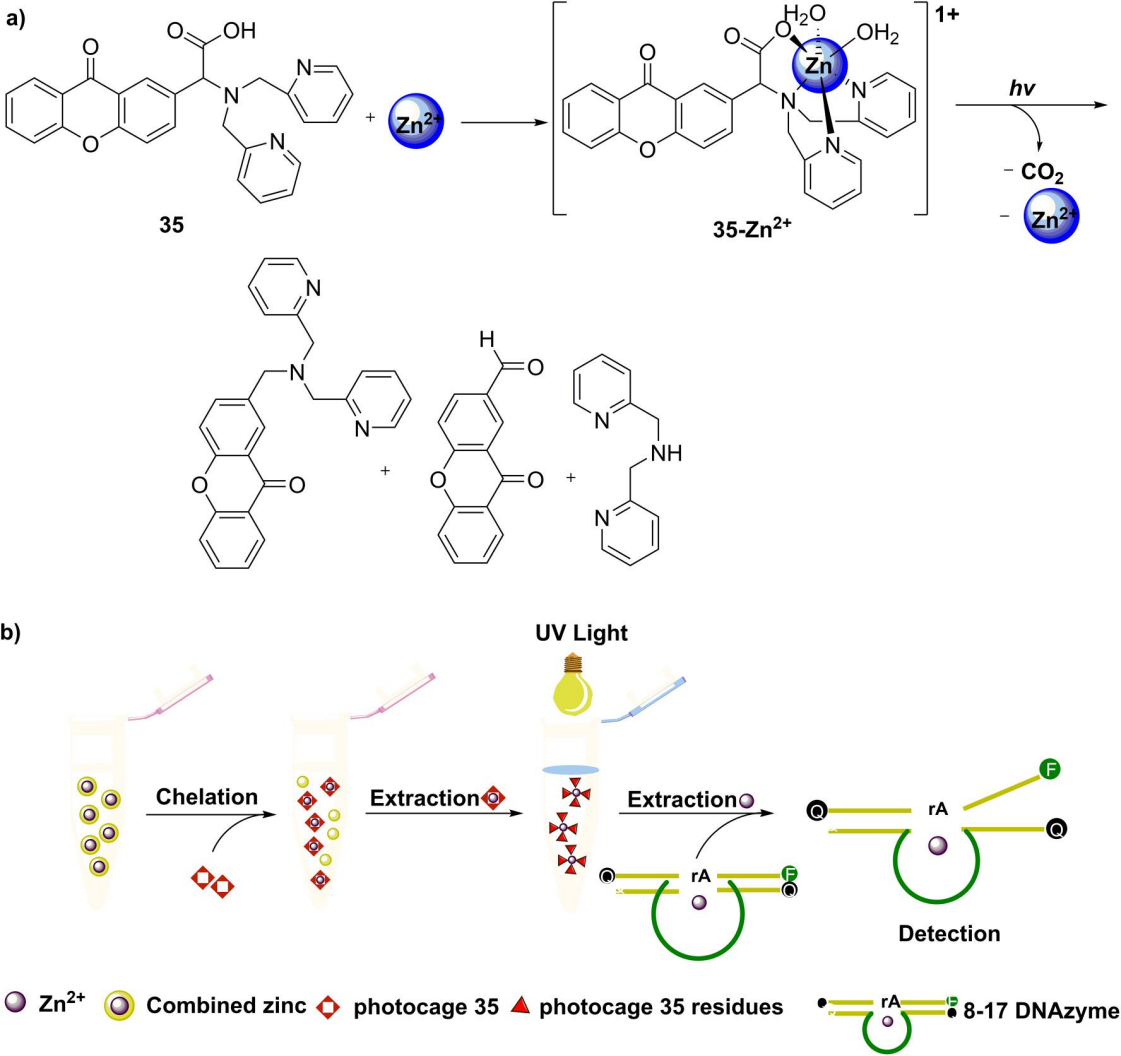
(a) Binding of Zn^2+^ by photocage **35** and the mechanism of photo‐controlled release of Zn^2+^ and (b) Scheme to chelate and extract Zn^2+^ from bovine newborn calf serum using photocage **35** and then the detection of Zn^2+^ using 8–17 DNAzyme.

Burdette's group was the first to develop metal ion‐releasing photocages based on the xanthene moiety. In 2010, this group developed photocage **33** (Figure 15) for the photo‐controlled release of Fe^3+^.^[36]^ Subsequently, in 2011, this group described photocage **34** (Figure 16) that releases Zn^2+^,^[37]^ an essential trace element that plays an extremely important role in humans. With an increase in interest in Zn^2+^ releasing xanthene‐based photocages, Burdette's group in 2019 developed photocage **35** (**XDPAdeCage**) that contains a xanthene‐9‐one derivative as the photosensitive group (Figure 17a)^[38]^ and has a 27% photolytic quantum yield. Using fluorescence imaging, they demonstrated the transport of Zn^2+^ across membranes. Additionally, they have used RT‐PCR techniques to measure the increased expression of metallothionein (MT) and zinc transporter, which are Zn^2+^‐responsive proteins in cells under irradiation. These two experiments have proved that photocage **36** can realize the intracellular release of Zn^2+^.

Despite many advances made, it is still challenging to detect and quantify metal ions that are tightly bound with biomolecules in serum. To address this problem, Burdette's group developed **XDPAdeCage** that tightly binds Zn^2+^ in serum and releases this ion under irradiation of 365 nm light; the release is quantified using 8–17 DNA enzyme (Figure 17b),^[39]^ which is a DNA metalloenzyme that catalyzes RNA transesterification in the presence of divalent metal ions.

**FIGURE 18:**
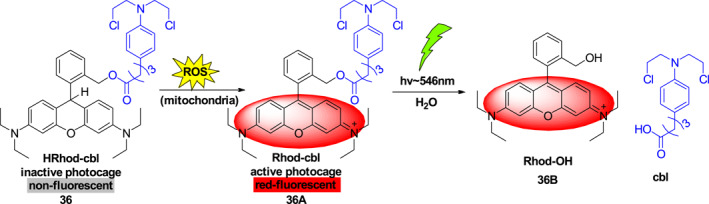
Photocage **36** and schematic of photo‐controlled release of chlorambucil.

Rhodamine, one of the most common xanthene derivatives,^[40]^ has been widely used in new photo‐controlled drug delivery systems.^[41]^ Photo‐controlled drug delivery systems (DDS) play important roles in biomedicine because they enable the precise release of encapsulated drugs spatiotemporally under specific stimulation conditions. The high level of drug utilization and low toxicity associated have made this strategy applicable to the precise treatment of major diseases such as tumors. In 2020, Singh et al. developed photocage **36** (Figure 18), possessing the antitumor drug chlorambucil (cbl) linked to a rhodamine fluorophore via an ester bond, for the photo‐controlled release of cbl.^[42]^ Photocage **36** in mitochondria of cancer cells is activated by oxidation with ROS to generate bright red fluorescing **36A**.^[43]^ Owing to the fact that most cancer cells produce 10 times more ROS than normal cells, **36A** accumulates mainly in cancer cells. Visible light irradiation at 546 nm promotes photolysis of **36A** to release cbl along with rhodamine fluorochrome **36B**, a red fluorescence substance that allows mitochondrial imaging to observe cells that contain the released drug. The developed rhodamine‐based photocage is an example of a new strategy for sensing, targeting and imaging, cells, and a promising platform for effective cancer diagnosis and treatment.

## 6 QUINONE AND DIARYLETHENE DERIVATIVE‐BASED PHOTOCAGES

**FIGURE 19:**
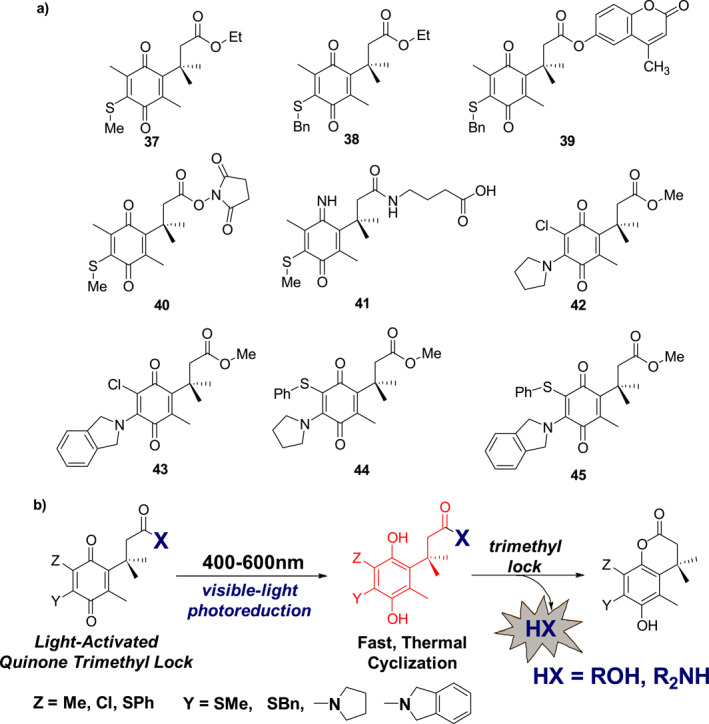
(a) The structures of photocages **37**–**45** based on the quinone trimethyl lock and (b) Mechanism of photoreactions of photocages **37**–**45**.

The “trimethyl lock” is a highly modular motif that has been found in wide applications as a protecting group for alcohols and amines.^[44]^ When a phenolic hydroxyl group is properly located near the trimethyl lock containing an ester or amide, rapid cyclization occurs to release ROH within seconds and R_2_NH within minutes, the process of which is commonly referred to as decaging. However, except for the trivial case of nitrobenzyl decomposition of phenol, no photochemically triggered trimethyl lock system had been reported in the studies carried out by Dougherty et al. until 2017. In this effort, a photochemically initiated trimethyl‐locked photocage system based on 1,4‐benzoquinone was developed. The quinone is reduced to phenol under visible light irradiation, followed by a trimethyl‐locked reaction that releases alcohol or amine from the photocages (Figure 19b).^[44]^ Using this strategy, Dougherty's group designed and synthesized a series of photocages **37–45** (Figure 19a), which can be used to release fluorescent agents and biologically related molecules. For example, photocage **39** rapidly releases a fluorescent sensor (hymecromone) in aqueous acetonitrile when irradiated at 455 nm. In a biological environment, photocage **41** releases the neurotransmitter γ‐aminobutyric acid (GABA) to activate GABA_A_ receptors in *Xenopus* oocytes upon irradiation at 455 nm.

**FIGURE 20:**
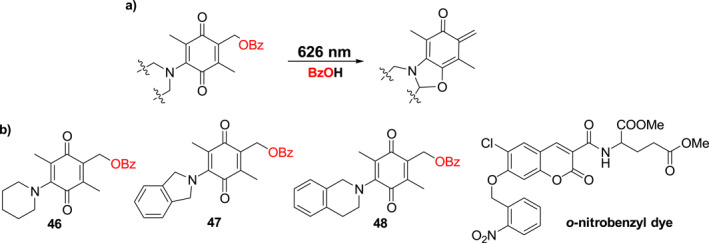
(a) Photoreaction mechanism and (b) the structures of photocages **46**–**48**.

The previously reported trimethyl lock photocages were activated by light with wavelength below 500 nm. To increase the activation wavelength range further into the red region, Kalow et al. designed the amino benzoquinone photocages **46**–**48** (Figure 20), containing two methyl groups and an amine moiety attached to the quinone core, are efficiently cleaved using 626 nm irradiation.^[45]^ The best‐performing photocage **48** has an 80% benzoic acid release rate in water/acetonitrile upon 626 nm LED irradiation. A qualitative comparison between the penetration depth of photocage **48** and that of *o*‐nitrobenzyl dye revealed that the newly reported photocage **48** has superior penetration depths. Due to their wavelength selectivity, high aqueous efficiency, low excitation energy, and good penetration depth, these photocages should have high utility for biological applications.

**FIGURE 21:**
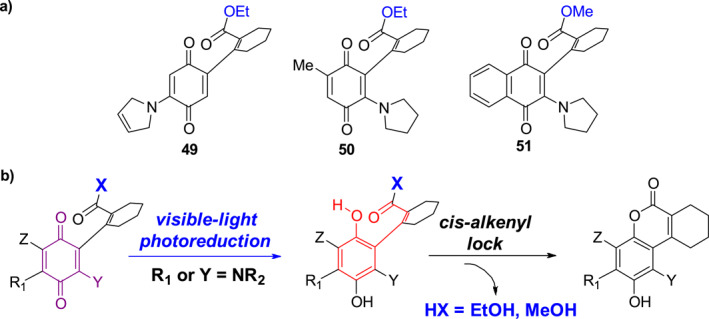
(a) The structures of photocages **49**–**51** and (b) Mechanism of photo‐controlled release of alcohols.

In another study aimed at overcoming the short irradiation wavelength problem, Dougherty et al. employed a *cis*‐lock strategy to design photocages **49**–**51** absorbing well above 450 nm (Figure 21a).^[46]^ Visible light‐promoted photolysis of the highly conjugated photocages **49**–**51** produces hydroquinones that rapidly undergo endo‐esterification to produce coumarin along with the release of alcohols (Figure 21b).

**FIGURE 22:**
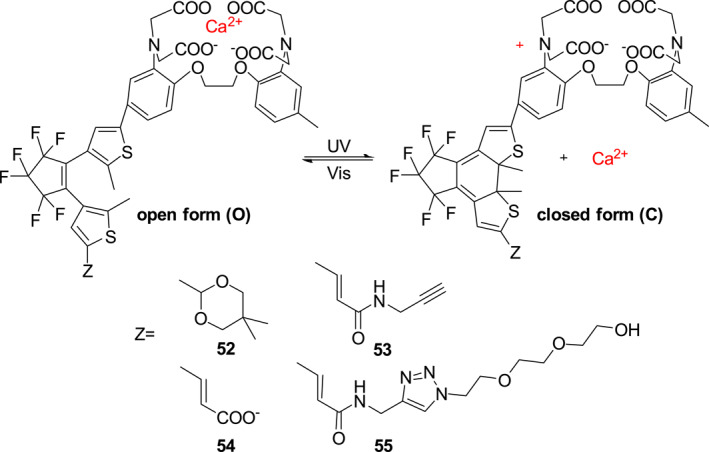
Wavelength‐controlled binding of photocages **52**–**55** with Ca^2+^.

In 2018, Plaza et al. described four photocages **52**–**55** that can be used for Ca^2+^ photo‐modulation (Figure 22).^[47]^ The photocages contain the 1,2‐bis (2‐aminophenoxy) ethane‐*N, N, N′, N′*‐tetraacetic acid, which is a well‐known Ca^2+^ ligand and diarylenes as photosensitive groups. Irradiation wavelength‐controlled electrocyclic diarylene ring opening and closing alters intramolecular charge transfer between N and the electron‐absorbing group (Z) on 1,2‐bis (2‐aminophenoxy) ethane‐*N, N, N′, N′*‐tetraacetic acid, which governs complexation and release of Ca^2+^.

**FIGURE 23:**
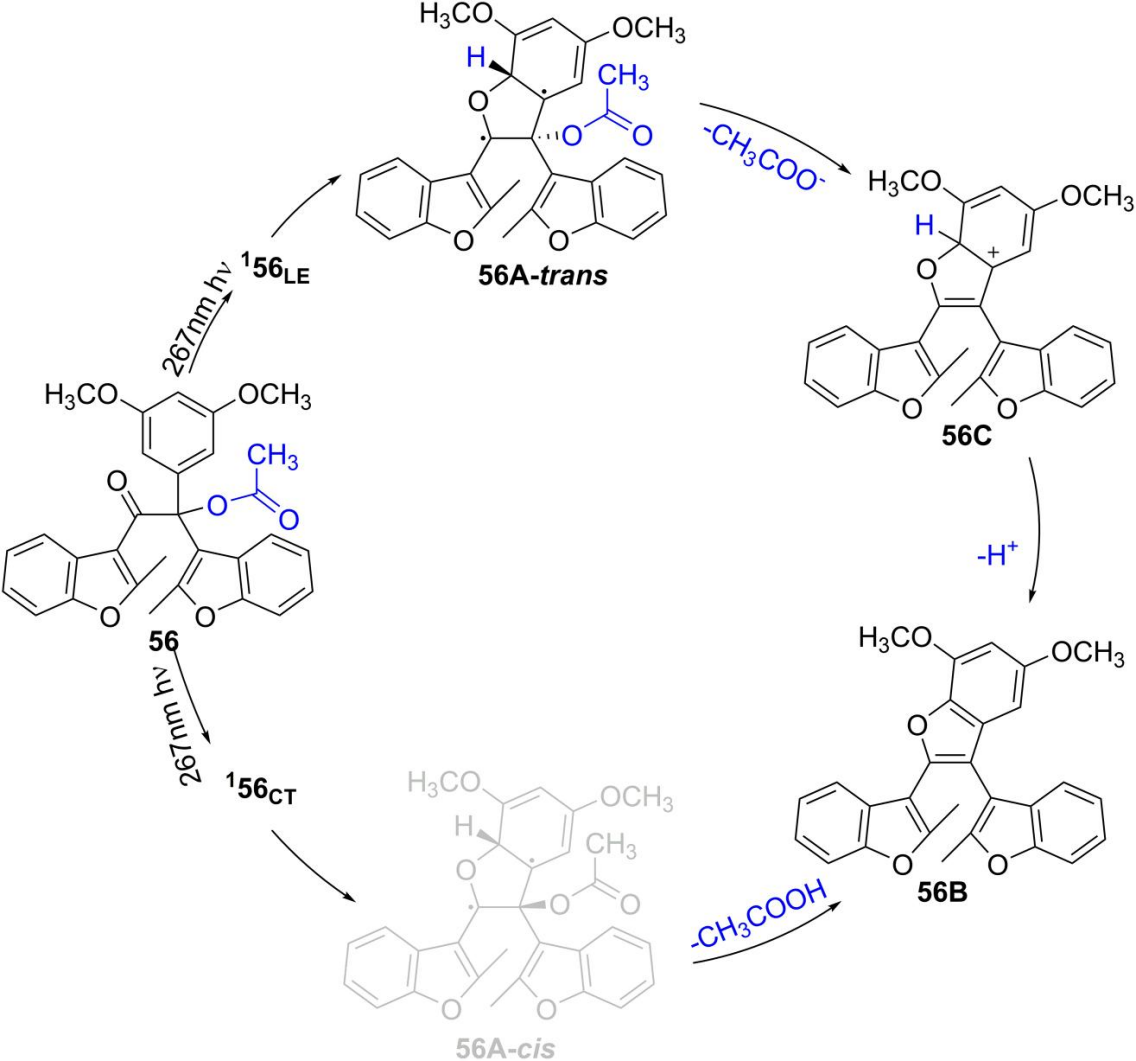
Photo‐deprotection mechanism of photocage **56**.

Although several literatures also reported novel photocage molecules consisting of dimethoxybenzoin group linked to diarylethylene derivatives, their photolytic mechanisms have not been studied in detail. Until 2022, Ma et al. synthesized 2‐acetoxy‐1,2,2‐tri (aryl) ethenone, photocage **56**, by introducing diarylethylene function into dimethoxybenzonin PPG platform (Figure 23) and investigated its photoprotection mechanism in depth.^[48]^ Under 267 nm light irradiation, photocage **56** is taken to the Franck–Condon region of the S_2_ state. Photocage **56** undergoes an internal conversion (IC) process to the first singly excited state (S_1_). In the Franck–Condon region, photocage **56** forms two different configurations of the singlet excited state (named ^**1**^ **56**_**LE**_ and ^**1**^ **56**_**CT**_). ^**1**^ **56**_**LE**_ was proposed to cyclize to produce **56A‐*trans***. **56A‐*trans*** further releases acetate to produce **56C** which can generate **56B** by subsequent deprotonation. In the other photocleavage route, ^**1**^ **56**_**CT**_ cyclizes to produce unstable **56A‐*cis***, then the concerted elimination of acetic acid leads to the production of **56B**. The declaration of this elaborate mechanism facilitates further design and improvement of diarylene‐based photoprotective moieties.

## 7 BIFUNCTIONAL PHOTOCAGES

**FIGURE 24:**
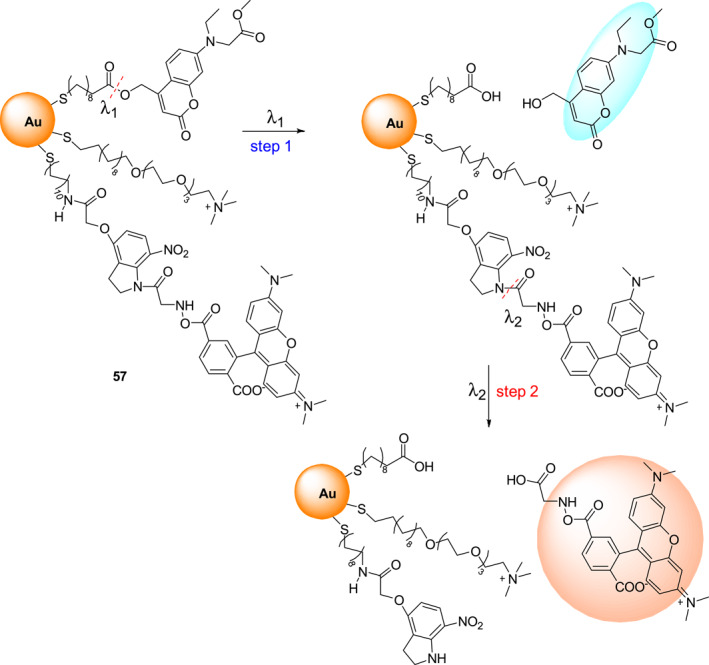
Sequential photocleavage of the AuNP‐SPC nanoparticle **57** by irradiation at 405 and then at 365 nm.

Bifunctional photocages contain two different photosensitive groups that can be selectively activated by irradiation at different wavelengths. Consequently, they are useful for carrying out two wavelength‐selective photo‐uncaging processes. Gold nanoparticles are a class of promising scaffolds for drug delivery, because they are biocompatible, easily synthesized, and have surface functional groups. In 2021, Zhou et al. developed an AuNP‐SPC nanoparticle system **57** using coumarinyl and 7‐nitroindolinyl as photosensitive groups (Figure 24).^[49]^ In this system, two different photosensitive groups can be selectively and progressively removed by varying the illumination wavelength. The ester bond in **57** is cleaved and the coumarin fluorophore is released preferentially upon irradiation at 405 nm, and then irradiation at 365 nm promotes cleavage of the amide bond with concomitant release of the rhodamine fluorophore. In addition, it was demonstrated that the AuNP‐SPC nanoparticle system **57** can undergo sequential, continuous, and selective irradiation induced release of the two fluorescent groups in living cells, so that this strategy provided a new platform for the probing of dynamic cellular systems.

**FIGURE 25:**
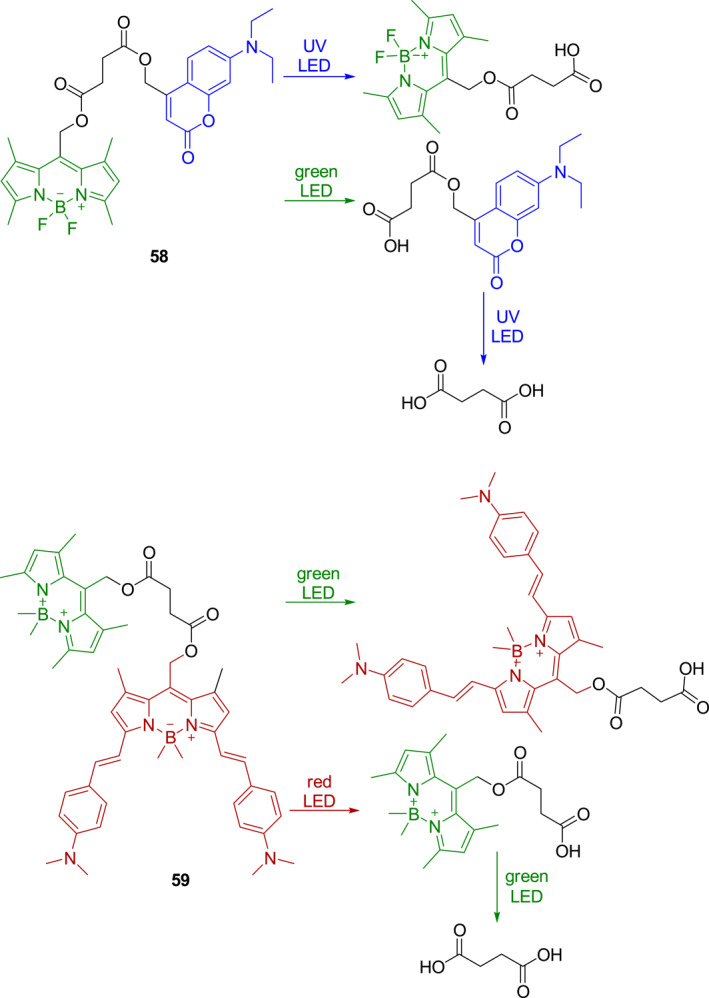
Photocages **58** and **59** and their photo‐uncaging mechanisms.

In 2021, Peterson et al. developed two bifunctional photocages **58** and **59** (Figure 25) that utilize coumarin and BODIPY derivatives as photosensitive groups.^[50]^ Both **58** and **59** undergo primary photolysis under irradiation at long wavelengths, accompanied by the release of their photosensitive moieties and corresponding fluorescence changes. Subsequently, secondary photolysis processes occur by irradiation at shorter wavelengths to release another fluorescence photosensitive moiety and succinic acid. It should be noted that the total photo‐uncaging process must be carried out by using sequential long‐ and then short‐wavelength irradiation, otherwise, no secondary process will take place. This method of selective deprotection of functional groups using different wavelengths of light is attractive for material synthesis and for achieving independent photo‐control of substrates in biological systems.

**FIGURE 26:**
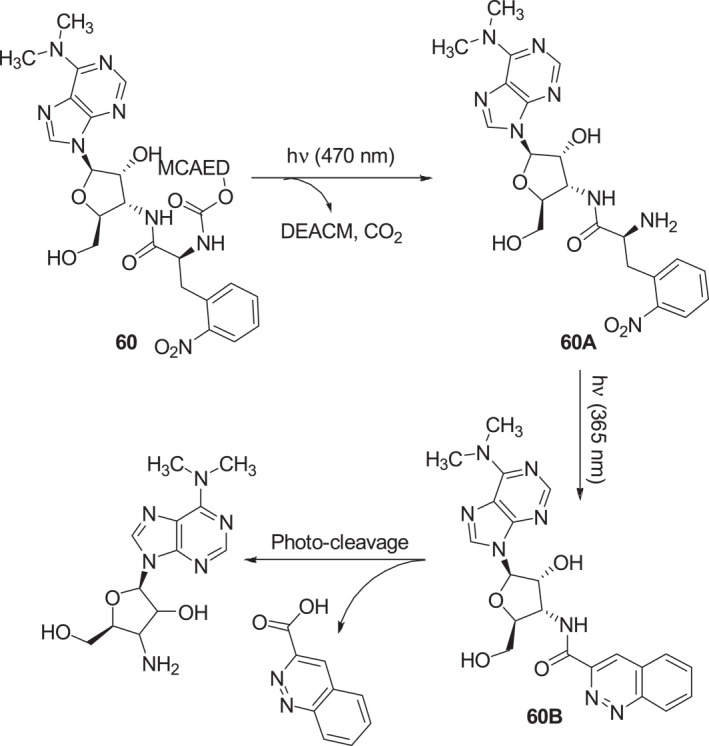
Dual functional photocage **60** and schematic of its mechanism of action.

In 2021, Schwalbe et al. described a wavelength‐selective sequential cleavage type photocage **60** that contains coumarin and *o*‐nitrobenzyl photosensitive groups (Figure 26).^[51]^ Photocage **60** can be activated by irradiation at 470 nm to release the coumarin derivative and form puromycin derivative **60A**, which inhibits shifted green fluorescent protein expression. **60A** undergoes a second photolytic reaction at the *o*‐nitrobenzyl center when irradiated at 365 nm to form the pyridazine ring in **60B** followed by cleavage of the amide bond and reversal of inhibition of protein expression. Photocage **60** has been used for wavelength‐selective inhibition of shifted green fluorescent protein expression in vitro. The approach to regulating activities of target biomolecules by using dual functional photocages is an important addition to methods for spatiotemporally controlling biological processes.

**FIGURE 27:**
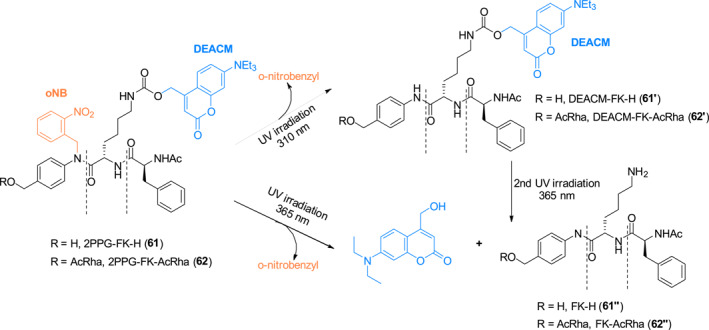
Schematic of sequential/direct photolysis of photocages **61** and **62**.

In 2022, Wang et al. reported a study that led to the development of the novel dual functional photocage **61** comprised of *o*‐nitrobenzyl and coumarin type photosensitive groups (Figure 27).^[52]^ Photocage **61** is very weakly fluorescent, probably due to photo‐induced electron transfer (PET) quenching of coumarin fluorescence by the *o*‐nitrobenzyl moiety. Under 310 nm irradiation (Figure 27), the *o*‐nitrobenzyl group in **61** was selectively removed to produce the intermediate DEACM‐FK‐H (**61′**), followed by the release of coumarin and FK‐H (**61″**) upon 365 nm light irradiation. In contrast, irradiation of **61** with 365 nm light promotes simultaneous removal of both PPG groups to produce FK‐H (**61″**). The authors also constructed photocage **62**, in which the cathepsin reporter acetyl rhodamine (AcRha) is linked to photocage **61**, for use to control and spatiotemporally track cathepsin activity by using 365 nm irradiation. The use of bifunctional photocages provides tighter control over the activity of probe by increasing its stability before decaging and introducing the possibility of continuous photo‐release. Additionally, since the substrate was installed in the final synthetic step, the cargo can be exchanged flexibly, so that the platform has great potential for switch probes and releasable prodrugs.

## 8 CONCLUSION

In summary, the studies described in this review show that great progress has been made in recent years in devising novel strategies to design photocages. These efforts have led to the development of photocages that are (i) based on various classes of photosensitive groups, (ii) more versatile and suitable for a wider range of applications, and (iii) more highly quantum efficient. Also, the wavelengths used to promote photo‐release from the photocages have been extended from the ultraviolet to the visible and near‐infrared ranges, which has led to reduced toxicities in biological systems and improved biocompatibility and permeability.

Importantly, novel photocages containing dual photosensitive groups have been developed not only to selectively control and track the activities of biomolecules, but also to create new platforms for probing the dynamics of cellular systems. However, the photosensitive groups in dual functional photocages need to have non‐overlapping absorption spectra to enable independent and selective promotion of two processes. As a result, the development of photocages containing dual photosensitive groups remains an important and practical challenge, and the construction of independently and selectively activated photocages is expected to be a goal of future research in this area.

## CONFLICT OF INTEREST STATEMENT

The authors declare no conflict of interest.
